# RIOK-1 Is a Suppressor of the p38 MAPK Innate Immune Pathway in *Caenorhabditis elegans*

**DOI:** 10.3389/fimmu.2018.00774

**Published:** 2018-04-17

**Authors:** Yi-Wei Chen, Wen-Chien Ko, Chang-Shi Chen, Po-Lin Chen

**Affiliations:** ^1^Institute of Basic Medical Sciences, College of Medicine, National Cheng Kung University, Tainan, Taiwan; ^2^Department of Internal Medicine, National Cheng Kung University Hospital, College of Medicine, National Cheng Kung University, Tainan, Taiwan; ^3^Department of Biochemistry and Molecular Biology, College of Medicine, National Cheng Kung University, Tainan, Taiwan; ^4^Department of Microbiology and Immunology, College of Medicine, National Cheng Kung University, Tainan, Taiwan

**Keywords:** *riok-1*, p38 MAPK/*pmk-1*, *skn-1*, innate immunity, immune suppressor, *Aeromonas dhakensis*, *Caenorhabditis elegans*

## Abstract

Innate immunity is the primary defense mechanism against infection in metazoans. However, aberrant upregulation of innate immune-signaling pathways can also be detrimental to the host. The p38 MAPK/PMK-1 innate immune-signaling pathway has been demonstrated to play essential roles in cellular defenses against numerous infections in metazoans, including *Caenorhabditis elegans*. However, the negative regulators that maintain the homeostasis of this important innate immune pathway remain largely understudied. By screening a focused RNAi library against the kinome of *C. elegans*, we identified RIOK-1, a human RIO kinase homolog, as a novel suppressor of the p38 MAPK/PMK-1 signal pathway. We demonstrated that the suppression of *riok-1* confers resistance to *Aeromonas dhakensis* infection in *C. elegans*. Using quantitative real time-PCR and *riok-1* reporter worms, we found the expression levels of *riok-1* to be significantly upregulated in worms infected with *A. dhakensis*. Our genetic epistasis analysis suggested that *riok-1* acts on the upstream of the p38 MAPK/*pmk-1* genetic pathway. Moreover, the suppression of *riok-1* enhanced the p38 MAPK signal, suggesting that *riok-1* is a negative regulator of this innate pathway in *C. elegans*. Our epistatic results put *riok-1* downstream of *skn-1*, which encodes a p38 MAPK downstream transcription factor and serves as a feedback loop to the p38 MAPK pathway during an *A. dhakensis* infection. In conclusion, *riok-1* is proposed as a novel innate immune suppressor and as a negative feedback loop model involving p38 MAPK, SKN-1, and RIOK-1 in *C. elegans*.

## Introduction

Coordination of the innate immune response in intestinal epithelial cells maintains host–microbial interaction and tissue homeostasis (1). In humans, overactivated or aberrantly triggered immune responses are associated with inflammatory diseases (2). The activation of the immune response is required for hosts to survive challenges from invading pathogens. By contrast, an inhibitory mechanism that functions to lessen the damage caused by pathogens limits collateral injury due to immune activation. For example, the ASK-1–MKK3/6–p38 MAPK signal pathway in mammals is a pivotal regulator of inflammatory cytokine production in response to pathogen detection at the mucosal surface, and the activation of the pathway is tightly regulated *via* negative regulatory circuits (3). The disruption of the p38 MAPK pathway has been closely associated with the pathogenesis of several human diseases, such as inflammatory diseases, cancers, and autoimmune diseases (3). Conceptually, immune tolerance to nonpathogenic bacteria may be beneficial for host survival. The coexistence of activation and suppression mechanisms for the immune response is like a balance between yin and yang to achieve homeostasis.

The p38 MAPK has evolutionarily conserved roles in the control of cellular responses to microbial and abiotic stress (3). The homolog of the ASK–1–MKK3/6–p38 MAPK signal pathway in mammals is NSY-1–SEK-1–PMK-1 MAPK in *Caenorhabditis elegans*. In the aging model, p38 MAPKK/SEK-1 is activated by NADPH oxidase-generated ROS, and the activation of the pathway promotes longevity in response to stress and is also negatively regulated by *memo-1* to maintain homeostasis (4). In addition, p38 MAPK/PMK-1-mediated resistance to bacterial infection has been well established in a *C. elegans* infection model (5). However, the regulatory mechanisms that ensure homeostasis of this pathway in response to infection are not very well understood. Previous studies have showed that a regulatory mechanism for p38 MAPK/PMK-1 pathway exists. MOM-4 has been reported to be an activator of the p38 MAPK immune pathway in response to *Pseudomonas aeruginosa* infection (6). By contrast, VHP-1, an MAP kinase phosphatase, is the only reported negative regulator of the p38 MAPK/PMK-1 innate immune pathway in *C. elegans* (7). We believe other unknown components that regulate the complex p38 MAPK/PMK-1 innate immune pathway exist and are yet to be discovered.

Kinases comprise one of the largest and most important protein families. The genes encoding kinases constitute 2% of genes in a variety of eukaryotic genomes and play major roles in modulating most cellular processes (8–10). The collection of kinases in an organism is termed the “kinome,” a concept that has been widely used to study life science from a general viewpoint (10, 11). The kinome of *C. elegans* has been reported to contain 418 protein kinases, 20 atypical kinases, and 25 kinase fragments or pseudogenes, which are about 80% homologous to human kinases (8, 9, 11). Here, we used the model organism *C. elegans* to study the immune response to a bacterial infection.

*Caenorhabditis elegans* is attractive as a model because of its suitability for studying innate immunity in a host (12, 13), conveniently related to gene analysis and observation, and its short life span. Moreover, it is rational to study immune homeostasis in organisms like bacterivorous *C. elegans* that live in microbe-rich environments and must defend against invading pathogens and tolerate food-source bacteria. In our study model, *A. dhakensis* was selected as an infectious pathogen *in vivo* because of its well-established virulence to *C. elegans* (14, 15) and, therefore, the presence of respective strategies by which to combat each other.

In the screening of the *C. elegans* kinome, we discovered that the loss of *riok-1* confers resistance to virulent *Aeromonas*, indicating its potential role as an immune repressor. RIOK-1, a human RIO (right open reading frame) kinase homolog that is universally present in both invertebrate and vertebrate animals and encodes an atypical protein kinase, which is known to participate in cell cycle control and ribosomal RNA processing (16–21). Previous studies have found that *riok-1* and *riok-2* are required for EGFR- and PI3K-mediated tumorigenesis in glioblastoma (22). However, the role of RIOK-1 in innate immunity against infection remains unclear. The present study further identified RIOK-1 as an immune suppressor that specifically regulates the p38 MAPK pathway.

In the present study, we identified *riok-1* as a novel innate immune suppressor and proposed a negative feedback loop model among p38 MAPK, SKN-1, and RIOK-1 in *C. elegans*. Bacterial infection activates the p38 MAPK pathway, which transcribes the expression of *riok-1* by *skn-1*, and the activation of *riok-1* results in downregulation of the p38 MAPK pathway.

## Materials and Methods

### Bacteria and *C. elegans* Strain

The *C. elegans* wild-type Bristol N2 strain, *riok-1*-mutant VC2676 *riok-1(gk1101)*, tissue-specific RNAi strain NL2099 *rrf-3(pk1426)*, NR222 *rde-1(ne219);kzIs9[lin-26p:nls:GFP* + *lin-26p:rde-1* + *rol-6(su1006)]*, VP303 *rde-1(ne219);kbIs7[nhx-2p:rde-1* + *rol-6(su1006)]*, TU3311 *uIs60[unc-119p:YFP* + *unc-119p:sid-1]*, and p38 MAPK pathway mutants, *sek-1(km4), pmk-1(km25)*, were provided by the Caenorhabditis Genetics Center (CGC). The p38 MAPK gain-of-function mutant *nsy-1(ums8)* was a gift from Dr. Read Pukkila-Worley (23). *riok-1* transcriptional reporter *riok-1p:mcherry* and translational reporter *riok-1p:riok-1:mcherry* were generated in this research. The animals were maintained on NGM plates using *Escherichia coli* strain OP50 as the food source. The *A. dhakensis* strain AAK1 used in the study was a clinical isolate from a patient with septicemia and necrotizing fasciitis (24). The *C. elegans* RNAi feeding clones were obtained from Ahringer’s *C. elegans* RNAi library and were kept in an RNAi vector pL4440 in *E. coli* strain HT115 (25).

### Screen of RNAi Kinome Library

The kinome RNAi library was collected from Ahringer’s *C. elegans* RNAi library followed by the *C. elegans* kinome database (8, 9, 11). Three hundred and four kinase RNAi clones from RNAi library were selected according to 438 *C. elegans* kinases. The excluded 134 kinases were either missing in the RNAi library or unable to culture. The RNAi clones were cultured in LB broth with 1 mM IPTG (to induce siRNA production), tetracycline, and ampicillin in 96 wells at 37°C overnight and were then mixed with 20–25 N2 L1 worms in 160 µl of M9 culture medium at 25°C for another 40 h. Ten microliters of *A. dhakensis* AAK1, which was incubated with LB broth at 37°C at a concentration of OD_600_ 2.0, was added to each well. The total numbers of live worms were counted after 2 days of culture at 25°C. The survival rates were calculated by measuring the numbers of live worms among the total worms as a percentage.

### Life Span Assay of *C. elegans* With RNAi

A plate assay was conducted to measure the life span of worms with bacterial infection. Briefly, the eggs were separated from the adult worms with a sodium hypochlorite/NaOH solution, and the resulting eggs were synchronized in M9 medium. The synchronized L1 larvae worms were seeded onto NGI plates (NGM plates with 1 mM IPTG) that were cultured with *E. coli* HT115 containing the desired RNAi target on the empty vector pL4440. The L1 worms grew at 20°C until the L4 stage. These L4 stage animals were transferred to plates together with *E. coli* HT115 either carrying RNAi plasmids or the empty vector together with *A. dhakensis* AAK1 at a ratio of 1:1. The animals were then incubated at 20°C. The worms were monitored for death events daily. Animals that escaped from the plate or died due to internal hatching were censored. Censored animals were included in the statistical analysis only until the day of the censoring event.

### Life Span Assay of *C. elegans* With Infection

Synchronized worms were grown on NGM plates covered with *E. coli* OP50 until the L4 stage. About 50 nematodes were transferred to fresh plates of OP50 or pathogens that were cultured in LB for 18 h at 37°C to achieve an OD_600_ 2.0 for tests. The worms were monitored for death events daily. Animals that escaped from the plate or died due to internal hatching were censored. Censored animals were included in the statistical analysis only until the day of the censoring event.

### Measurement of Gene Expression

The expression of *riok-1*, p38 MAPK downstream genes, and *nhr-23* as an internal control was measured using quantitative real-time-PCR (qRT-PCR) as described previously (15). Approximately 2,000 worms were collected for RNA extraction. An RNA sample (2.0 µg) for each experimental group was converted to cDNA *via* reverse transcription. All qRT-PCRs were carried out using the FastStart Universal SYBR Green Master (Rox) according to the manufacturer’s specifications and analyzed on a StepOnePlus Real-Time-PCR System. The expression level of each target gene was collected as a ΔCt value, where Ct was equal to the number of PCR cycles required to amplify a given gene from a cDNA population. The fold-change values were estimated using the following equation: fold change = 2^[−ΔCt (AAK1)]^/2^[−ΔCt (OP50)]^. Changes in the expression genes were initially measured as ΔCt values which were subsequently normalized against a housekeeping gene *nhr-23*. Primers for qRT-PCR included *riok-1* (forward primer: CGA AAG ATT GCT ATG CAC ACG; reverse primer: CTT CCT CTG TTC CCG TTT CTC), *riok-1* isoform a (forward primer: CCG GCT CCG TTG CTT AAA; reverse primer: TCT CAT ATC GCG CAC CAA AC), *riok-1* isoform b (forward primer: GGC CAT GTA CTT GTG ATG GA; reverse primer: TCT CAT TTA TCC ACA CCT CTT GG), K08D8.5 (forward primer: GAA TCT TTC GGA GCC CTA CTA C; reverse primer: CGT TCC CTG AGG AAC ATT GA), C32H11.12 (forward primer: CGA GCC AGG AGG TTA TCT TTA C; reverse primer: GTC CGT CCC GAT GTT GAT TT), C29F3.7 (forward primer: CGT ATC TTG GAA CAG GAC TTC A; reverse primer: CAG CCC AGG AAT CAC CAA TA), F08G5.6 (forward primer: TGT CCA TTT CCA TCG CAT ACT; reverse primer: CGG TAG ATT GCT AAT GGG TTC T), T24B8.5 (forward primer: TGT AAC GAA GCA GAT GTT AGA AGT G; reverse primer: TGG CTC TGC AGT TGT ACC A), *nhr-23* (forward primer: GCC GAA GAT GAT GCC GAG AT; reverse primer: GTC GCA GTG TCA AGA ATC CC).

### Construction of the *riok-1* Reporter

To create *riok-1p:mCherry* and *riok-1p:mCherry:riok-1*, a 2,000-bp region 5' occupying the ATG site and the *riok-1* cDNA fused with mCherry (pCG150) were constructed and then cloned into pPBCN39 using the BP/LR gateway system. *unc-119* worms were microinjected, and the offspring of movers with florescence were picked and confirmed with sequencing.

### Tissue-Specific RNAi

Synchronized NL2099, NR222, VP303, and TU3311 L1 larvae were cultured on plates seeding *E. coli* HT115 with L4440, *riok-1* RNAi, and tissue-specificity control RNAi *act-5, unc-73*, and *bli-1* at 20°C until the L4 stage. RNAi knockdowns were restricted specifically in whole body (NL2099), epidermis (NR222), intestine (VP303), and neurons (TU3311). These L4 stage animals were transferred to plates together with *E. coli* HT115 either carrying RNAi plasmids or the L4440 plasmid together with *A. dhakensis* AAK1 at a ratio of 1:1. The animals were then incubated at 20°C and the survival was observed.

### p-p38 Western Blot

Analysis of the p38 MAPK activation of *C. elegans* was performed using a Western blot method described previously with some modifications (26). Briefly, approximately 2,000 worms were collected to extract the total protein with a RIPA buffer containing inhibitors for protease and phosphatase. Monoclonal antibody to phospho-p38 MAPK (Cell Signaling Technology) and monoclonal antibody to tubulin (Sigma-Aldrich) were used for detection.

### Statistical Analysis

All experiments were performed a minimum of three times independently. Nematode survival assays were assessed using the Kaplan–Meier method, and survival differences were tested using the log-rank test. The results of the statistical analysis between two values were compared with a Student’s *t*-test. Statistical analysis among three or more values for one independent variable was done with a matched one-way ANOVA test. Statistical significance was set at *P* < 0.05.

## Results

### Suppressing Expression of *riok-1* Confers Resistance Against *A. dhakensis* Infection in *C. elegans*

**Figure 1:**
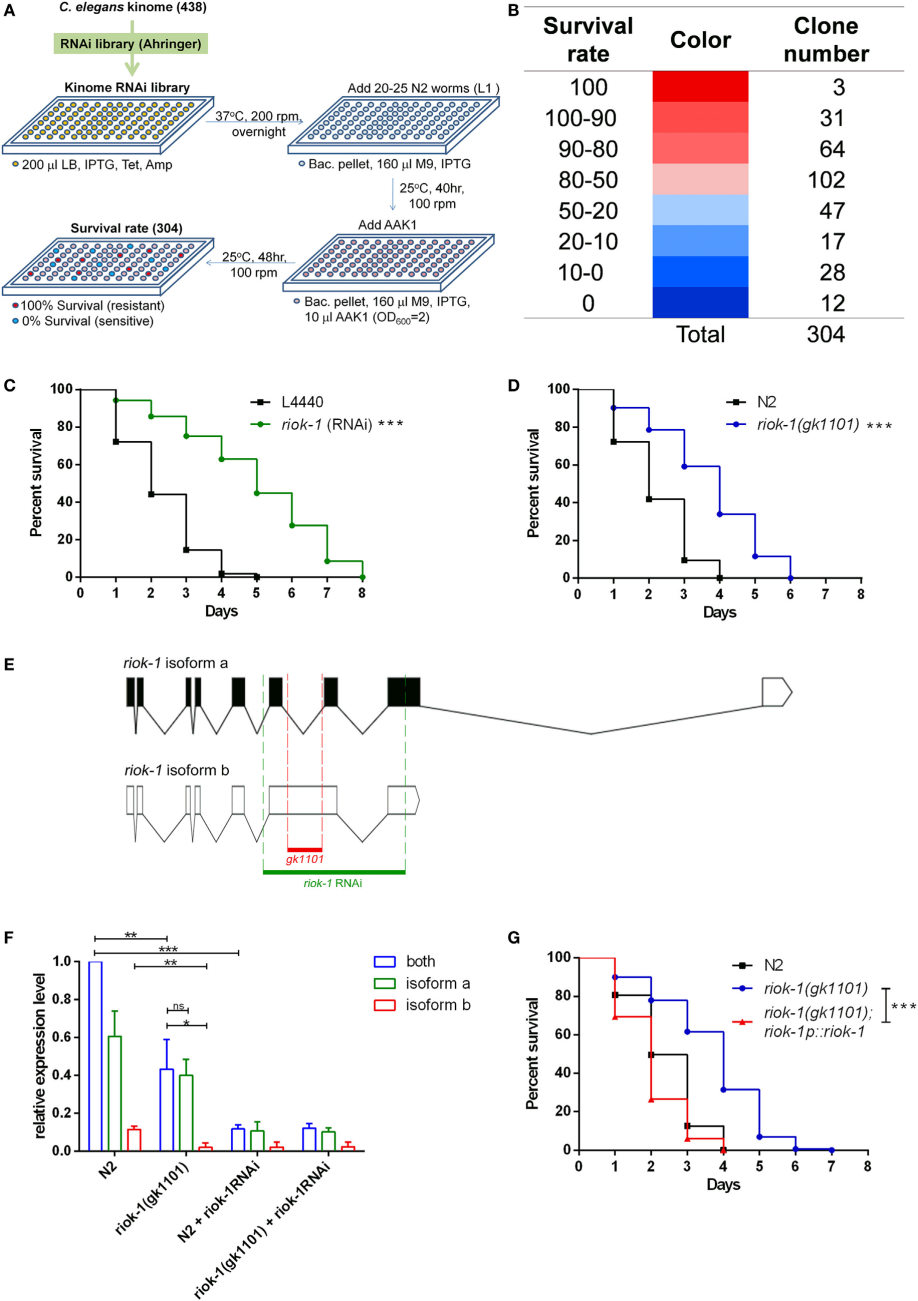
Loss of *riok-1* confers resistance to *Aeromonas dhakensis* infection in *Caenorhabditis elegans* through screening an RNAi kinome library. Through screening the RNAi kinome library in *C. elegans*, worms with *riok-1* knockdown showed resistance to *A. dhakensis* infection. **(A)** Flowchart showing the screening of the RNAi kinome library in *C. elegans*. Three hundred and four kinase RNAi clones from the RNAi library were selected according to 438 *C. elegans* kinases. The excluded 134 kinases were either missing in the RNAi library or unable to culture. **(B)** Hit numbers at each level classified by the survival rate of worms in the RNAi kinome library. **(C)** Compared to worms with empty vector control L4440, worms with RNAi-mediated knockdown of *riok-1* showed resistance to *A. dhakensis* (***P < 0.001). **(D)** *riok-1(gk1101)* worms showed resistance to *A. dhakensis* compared to wild-type N2 worms (***P < 0.001). **(E)** The location of *riok-1(gk1101)* (in red) and the target region of *riok-1* RNAi (in green) labeled on *riok-1* isoforms. **(F)** Expression level of *riok-1* isoforms by quantitative real time-PCR (qRT-PCR) in wild-type N2 worms and *riok-1(gk1101)*-mutant worms with or without treatment with *riok-1* RNAi (***P < 0.001, **P < 0.01, and *P < 0.05). **(G)** The complement of *riok-*1 in *riok-1 (gk1101)* mutants reversed the resistant phenotype of *riok-1(gk1101)* worms (***P < 0.001). IPTG, isopropyl β-D-1-thiogalactopyranoside 1 mM; Tet, tetracycline 15 µg/ml; Amp, ampicillin 50 µg/ml; M9, worm M9 buffer.

We created a focus RNAi library (Figure 1A) of the *C. elegans* kinome according to the kinome database (8, 9, 11). Our library contained 304 kinase RNAi clones that covered 69.41% of currently known kinases (Figure 1B; Table S1 in Supplementary Material). We knocked down the expression of kinases using specific RNAi and observed their influence on the survival of worms with *A. dhakensis* infection. The results were grouped with the survival rate of worms and are partially listed in Tables 1A,B. The screening results were compatible with previous findings suggesting that the repression of kinases, such as *aak-1* (27), *dkf-2* (28), and *sek-1* (5, 29), participates in immune pathway-altered survival shortening of worms with *Aeromonas* infection (Table 1B). Among these kinases, the repression of *riok-1*, one of the atypical serine kinases known to be associated with rRNA maturation, significantly improved the resistance of *C. elegans* with *A. dhakensis* infection (Table 1A). RIOK-1 was conserved from *C. elegans* to mammals (17), and its activation led to tumorigenesis in glioblastoma cancer cells as reported in Ref. (22). The N2 worms treated with *riok-1* RNAi enhanced the resistance to *A. dhakensis* strain AAK1 in the plate-killing assay (Figure 1C). The *riok-1* knockdown worms were significantly resistant to *A. dhakensis* in comparison to wild-type worms (*P* < 0.001). A previous study showed that different assays may result in different outcomes in a *C. elegans* infection model (30). Our study showed similar survival results in the plate assay (Figure 1C) and the screening liquid assay (Table 1A; Table S1 in Supplementary Material), showing that worms with *riok-1* suppression mediated by RNAi were more resistant to *A. dhakensis* infection.

**Table 1:** Excerpt from the kinome RNAi screen.

A	Sequence name	Gene	Survival rate	SD	*p*-Value
RNAi control vector pL4440			28.39	6.67	–
Gene depression confers resistance to *Aeromonas. dhakensis*	M01B12.5	*riok-1*	100.00	0.00	<0.001
	C04G2.2		100.00	0.00	<0.001
	C01H6.9	*hasp-1*	100.00	0.00	<0.001
	R06C7.8	*bub-1*	98.81	2.06	<0.001
	B0511.4	*tag-344*	98.41	2.75	<0.001
	ZC404.9	*gck-2*	98.33	2.89	<0.001
	Y54E10B_152.b	*mek-2*	98.15	3.21	<0.001
	T01H8.1	*rskn-1*	97.62	2.20	<0.001
	T09B4.7		97.60	2.21	<0.001
	F42A10.4	*efk-1*	97.29	2.53	<0.001
	K06H7.1		96.97	5.25	<0.001
	F46F5.2		96.93	2.95	<0.001
	Y106G6A.1		96.90	2.69	<0.001
	F59A3.8		96.83	5.50	<0.001
	Y65B4A_185.a		96.30	6.42	<0.001
	F23C8.7		96.00	6.93	<0.01
	F58A3.2	*egl-15*	95.49	4.78	<0.001
	Y48G1B_55.a		95.42	5.39	<0.001
	F52F12.3	*mom-4*	95.24	8.25	<0.01
	F35G12.3	*sel-5*	95.24	8.25	<0.01
	C34F11.5		95.15	5.69	<0.001
	T13H10.1	*kin-5*	94.48	3.53	<0.001
	F23C8.8		93.94	10.50	<0.01
	K03E5.3	*cdk-2*	92.64	2.55	<0.001
	K06H7.8		92.50	6.61	<0.001
	F46F6.2	*pkn-1*	91.83	7.08	<0.01
	C05C12.1		91.44	5.79	<0.001
	F49B2.5	*src-2*	91.07	6.89	<0.01
	F59A6.4		90.97	12.06	<0.01
	K08H2.5		90.77	7.03	<0.01
	F26E4.5		90.70	3.31	<0.001
	W04G5.6	*kin-23*	90.48	16.50	<0.05
	C44C8.6	*mak-2*	90.45	8.29	<0.01
	Y38F1A.10	*max-2*	90.14	7.80	<0.01

**B**	**Sequence name**	**Gene**	**Survival rate**	**SD**	***p*-Value**

RNAi control vector pL4440			28.39	6.67	–
Gene depression confers resistance to *A. dhakensis*	R03G5.2	*sek-1*	9.87	6.54	<0.05
	F09A5.2		9.76	1.17	<0.001
	F22D6.5	*prpf-4*	9.18	7.95	<0.05
	C25A8.5		9.17	5.46	<0.05
	F57B9.8		8.38	7.70	<0.05
	C08H9.5	*old-1*	7.34	6.53	<0.05
	C50H2.7		7.14	3.89	<0.01
	F16B12.5		6.71	5.97	<0.05
	F52B5.2		6.56	6.92	<0.05
	D2023.6		6.55	2.22	<0.001
	F12F3.2		6.06	10.50	0.058
	R11G1.4	*sax-1*	5.68	5.89	<0.01
	C07G1.3	*pct-1*	5.33	6.84	<0.05
	B0218.5		5.00	8.66	<0.05
	C34G6.5	*cdc-7*	4.89	4.84	<0.01
	F55C5.7	*rskd-1*	4.44	7.70	<0.05
	W07G4.3		4.29	4.17	<0.01
	C49C8.1		4.17	4.17	<0.01
	F22B3.8		4.07	3.57	<0.001
	R90.1		2.56	4.44	<0.01
	T19A5.2	*gck-1*	2.47	4.28	<0.01
	F26A1.4		2.15	3.72	<0.001
	PAR2.3	*aak-1*	1.67	2.89	<0.001
	F49C5.4		1.67	2.89	<0.001
	F13B9.4		1.08	1.86	<0.001
	C24G6.2		1.04	1.80	<0.001
	T25B9.4		0.95	1.65	<0.001
	B0496.3	*unc-82*	0.93	1.60	<0.001
	ZC449.3	*sek-3*	0.00	0.00	<0.001
	Y39H10A_224.a	*chk-1*	0.00	0.00	<0.001
	Y39G8B.f		0.00	0.00	<0.001
	W08D2.8	*kin-21*	0.00	0.00	<0.001
	T25E12.4	*dkf-2*	0.00	0.00	<0.001
	R02C2.2	*kin-34*	0.00	0.00	<0.001
	F57F5.5	*pkc-1*	0.00	0.00	<0.001
	F49E11.1	*mbk-2*	0.00	0.00	<0.001
	F47F2.1		0.00	0.00	<0.001
	F11D5.3	*ddr-2*	0.00	0.00	<0.001
	F09G2.1		0.00	0.00	<0.001
	C44C10.7		0.00	0.00	<0.001

*The table shows a section of the screening results of the kinome RNAi. For the full screening results of the kinome RNAi, please see Table S1 in Supplementary Material*.

*(A) The RNAi knockdown targets of kinase that caused higher survival rates in A. dhakensis strain AAK1-infected C. elegans*.

*(B) The RNAi knockdown targets of kinase that caused lower survival rates in A. dhakensis strain AAK1-infected C. elegans*.

The resistance to *A. dhakensis* in *riok-1(gk1101)*-mutant worms was similar to that observed in worms treated with *riok-1* RNAi (*P* < 0.001) (Figure 1D). Of note, we found the life span of *riok-1* mutant to be shorter than that of *riok-1* knockdown worms under *A. dhakensis* infection. It seems that worms with *riok-1* knockdown are more resistant to *A. dhakensis* infection than with *riok-1* mutants. The difference may be explained by the fact that the *riok-1(gk1101)* worm is not a *riok-1* null mutant that still maintains isoform a, one of the two isoforms of *riok-1* (Figure 1E). The resistance of worms with *riok-1* knockdown by RNAi is stronger than that of *riok-1(gk1101)* worms, which is correlated with the lower-expression level of *riok-1* in worms measured by qRT-PCR (Figure 1F). The expression level of riok-1 isoform b was significantly lower in *riok-1(gk1101)* than in wild-type N2 worms (*P* < 0.01). Nevertheless, overexpressed *riok-1* in the *riok-1*(*gk1101*) mutant reversed the resistant phenotype of the *riok-1(gk1101)* mutant upon *A. dhakensis* infection (Figure 1G). Taken together, these results showed that suppressed *riok-1* expression confers resistance against *A. dhakensis* infection in *C. elegans*.

### Expression Level of *riok-1* Is Increased Under *A. dhakensis* Infection in *C. elegans*

**Figure 2:**
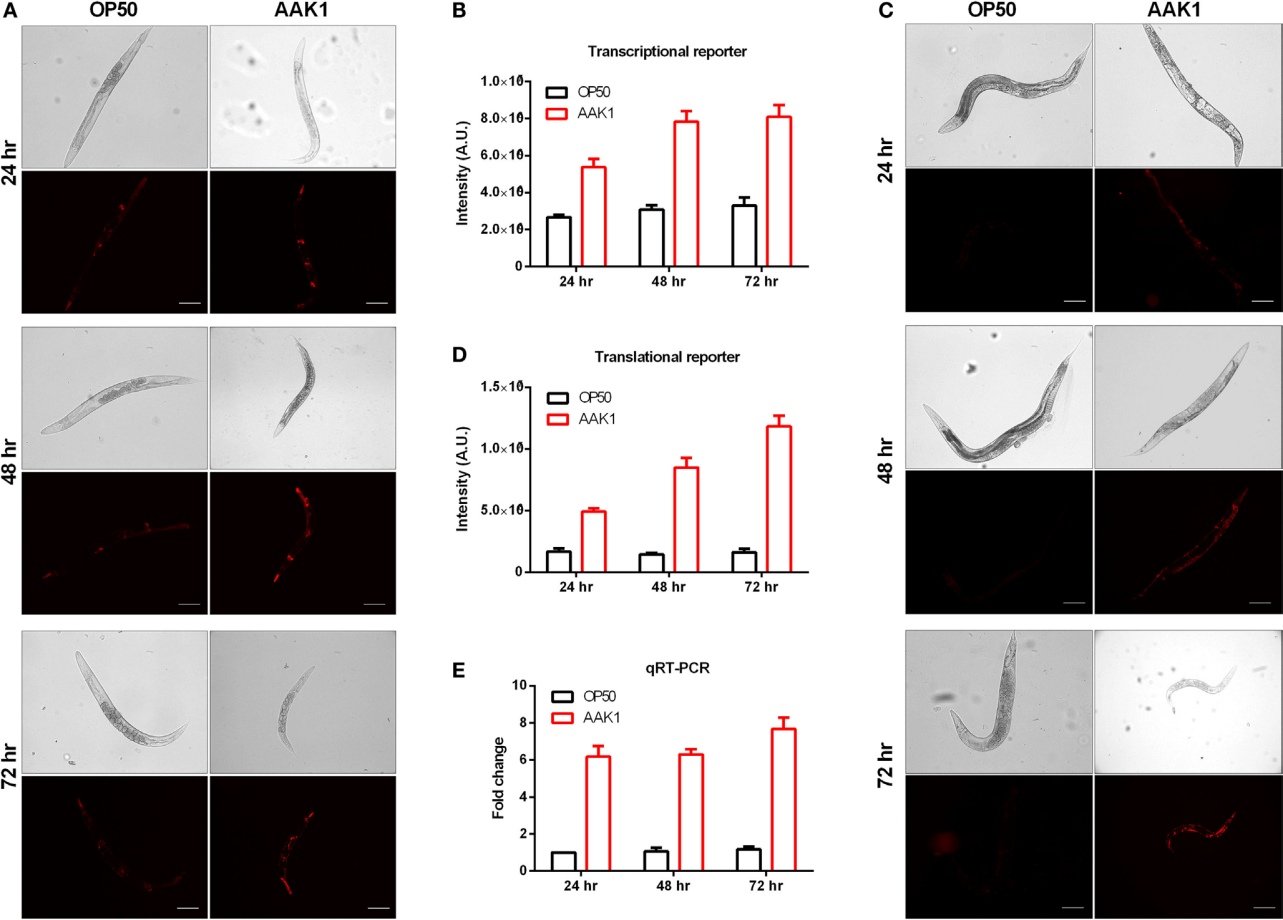
Expression level of *riok-1* induced upon *Aeromonas dhakensis* infection. **(A)** *riok-1* expression in the *riok-1* transcriptional reporter strain (*riok-1p:mCherry*) **(B)** Quantification of *riok-1* expression in *riok-1* transcriptional reporter worms. **(C)** *riok-1* expression in the translational reporter strain (*riok-1p:mCherry:riok-1*) **(D)** Quantification of *riok-1* translation in the reporter strain. **(E)** *riok-1* level as determined by quantitative real time-PCR (qRT-PCR). The scale bars in **(A,C)** are all 100 µm. The statistical analyses of OP50 and AAK1 at each time point in **(B,D,E)** are all *P* < 0.001.

In order to monitor the expression level and pattern of *riok-1* in *C. elegans*, we generated the *riok-1* transcriptional reporter (*riok-1p:mCherry*) and translational reporter (*riok-1p:mCherry:riok-1*), respectively. In these *riok-1* reporter worms, *riok-1* expression was increased in a time-dependent manner after *A. dhakensis* was added (Figures 2A,C). The *riok-1* transcriptional and translational levels were compatible and are summarized in Figures 2B,D. The expression level of *riok-1* as measured by qRT-PCR is also compatible with those observed in reporter worms microscopically (Figure 2E). Taken together, *A. dhakensis* AAK1 infection can induce *riok-1* expression in *C. elegans*.

### Knockdown of *riok-1* in Intestine and Neurons Conferred Resistance to *A. dhakensis* in *C. elegans*

**Figure 3:**
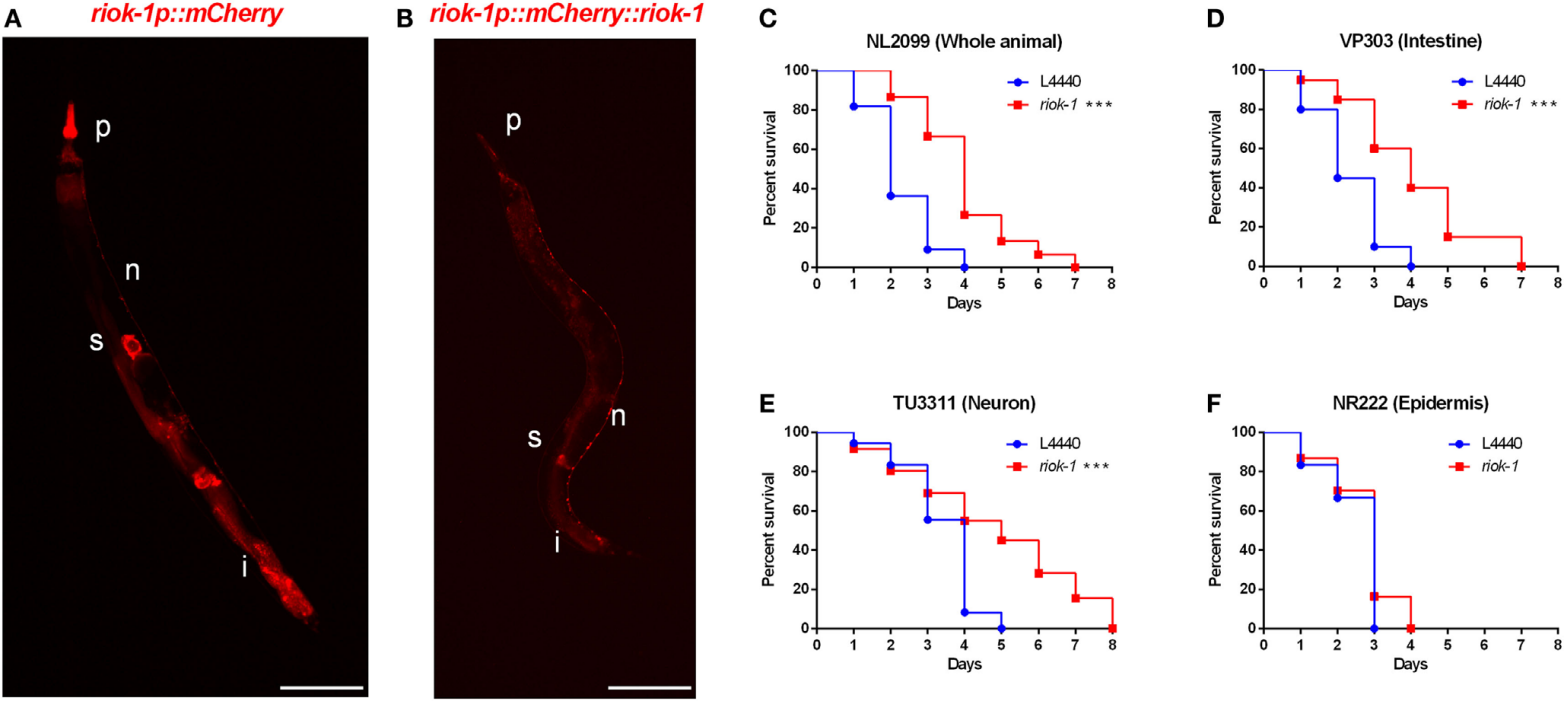
Tissue-specific knockdown of *riok-1* in the intestines and neurons resulted in similar resistance to *Aeromonas dhakensis* as in worms with general *riok-1* knockdown. **(A)** *riok-1* is expressed in the pharynx (P), intestines (i), spermatheca (s), and pan-neuronally (n) in *riok-1* transcriptional reporter worms. **(B)** *riok-1* is expressed in the pharynx (p), intestines (i), spermatheca (s), and pan-neuronally (n) in translational reporter worms. **(C–F)** *A. dhakensis* resistance was seen in worms infected by A. *dhakensis* AAK1 with tissue-specific knockdown of *riok-1* in the whole body **(C)**, intestines **(D)**, and neurons **(E)** in contrast to non-resistance to *A. dhakensis* among those with *riok-1* knockdown in the epidermis **(F)** ***P < 0.001. The scale bars in **(A,B)** are both 100 µm.

We found that the expression of *riok-1* was increased by *A. dhakensis* infection. The constitutive expression of *riok-1* appeared in specific parts of *C. elegans*. Our data showed that *riok-1* is expressed in many neurons, in the intestine, in the pharynx, and probably in spermatheca in the *riok-1* transcriptional reporter worms (*riok-1p::mCherry*) (Figure 3A; Figures S2A, S2C, and S2D in Supplementary Material) as well as the translational reporters (*riok-1p::mCherry::riok-1*) (Figure 3B; Figure S2B in Supplementary Material), as described in previous reports (31, 32). In order to determine the site of action of *riok-1* and clarify the tissue specificity of *riok-1* in terms of *A. dhakensis* resistance, the life span assay of worms treated with *riok-1* RNAi restricted to specific tissues was observed. The specificity of tissue-specific RNAi worm strains was confirmed with tissue-specific RNAi clones, which acted only in the corresponding tissues and demonstrated specific phenotypes (33) (Figure S2E in Supplementary Material). With tissue-specific RNAi worms, the knockdown of *riok-1* in the whole body (Figure 3C), intestine (Figure 3D), and neurons (Figure 3E) enhanced resistance to *A. dhakensis* infection in *C. elegans*. By contrast, suppressed *riok-1* expression in the epidermis did not alter the life span of worms with *A. dhakensis* AAK1 infection (Figure 3F).

### Progeny Deficiency Is Not Associated With Resistance to *A. dhakensis* Infection Mediated by Suppressing *riok-1*

Previous studies have shown that germ-cell loss extends the life span of *C. elegans* (34, 35). Our observation was compatible with previous findings, showing that *riok-1* deletion confers progeny reduction (32). To clarify whether the progeny deficiency caused by *riok-1* RNAi or the *riok-1* mutant confers longer life span in *A. dhakensis* infection, *glp-4(bn2)* worms were tested. The *glp-4* gene is critical for germ line proliferation, and *glp-4(bn2)* worms are characteristic of germ line loss with progeny deficiency at 25°C. Gaining resistance from loss of fertility against pathogen infection has been widely reported (36). Nevertheless, our results showed that the survival curve of *glp-4(bn2)* worms infected by *A. dhakensis* was similar to that observed among N2 worms with or without *riok-1* knockdown mediated by RNAi (Figure S3 in Supplementary Material). These findings suggest that progeny deficiency is not associated with *A. dhakensis* resistance mediated by *riok-1* suppression in *C. elegans*.

### RIOK-1 Is a Suppresser of the p38 MAPK Immune Pathway

**Figure 4:**
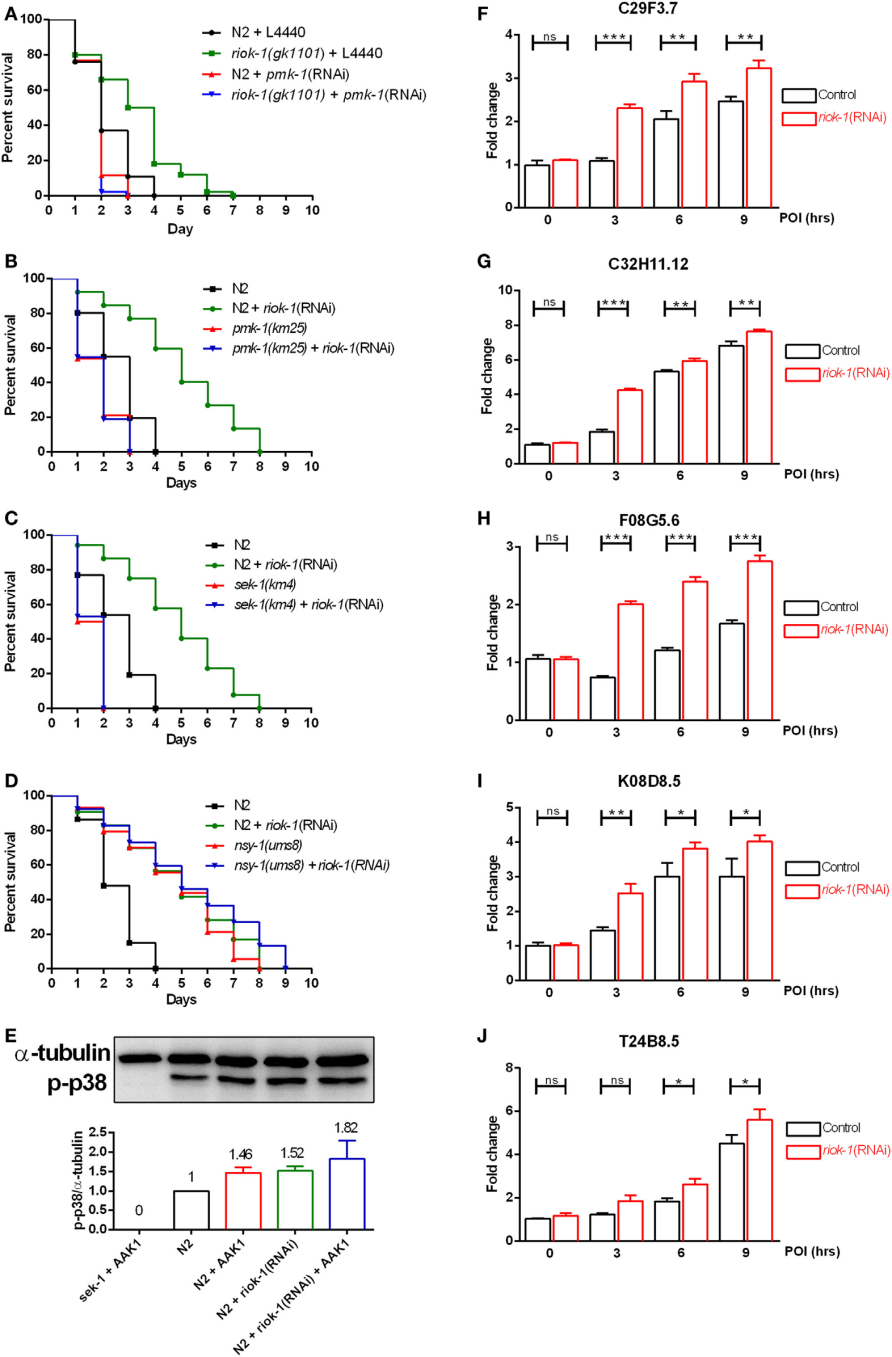
*riok-1* negatively regulates the p38 MAPK pathway. **(A)** Life spans of wild-type N2 and *riok-*1(*gk1101*) worm knockdown with *pmk-1* RNAi were both shortened. **(B)** MAPK (*pmk-1*) mutants, **(C)** MAPKK (*sek-1*) mutants, and **(D)** MAPKKK (*nsy-1*) gain-of-function mutants with *riok-1* knockdown using RNAi showed similar life spans when compared with those without RNAi knockdown under *A. dhakensis* infection. **(E)** *A. dhakensis* infection and RNAi-mediated *riok-1* knockdown induced p38 phosphorylation as shown in a Western blot analysis. **(F–J)** Increasing expression of p38 MAPK downstream genes C29F3.7 **(F)**, C32H11.12 **(G)**, F08G5.6 **(H)**, K08D8.5 **(I)**, and T24B8.5 **(J)** as determined using qRT-PCR in worms with *riok-1* knockdown mediated by RNAi under *A. dhakensis* infection. For the unprocessed data of western blot analysis in **(E)**, please see the Figure S6 in Supplementary Material. ***P < 0.001, **P < 0.01, and *P < 0.05.

Our results showed that progeny deficiency did not contribute to *A. dhakensis* resistance in *riok-1*-deficient worms (Figure S3 in Supplementary Material). We also found tissue-specific knockdown of *riok-1* in the intestine and neurons, which are two major organs where innate immunity present in *C. elegans* increased the resistance to *A. dhakensis* infection (Figures 3D,E). We then hypothesized that *riok-1* may regulate innate immunity in *C. elegans*. In order to understand the relationship between *riok-1* and innate immunity in *C. elegans*, the major innate immune-signaling pathways were screened. The screening showed that worms with suppressed *riok-1* expression had increased susceptibility upon *A. dhakensis* infection when *pmk-1* was downregulated with RNAi, which was similar to the susceptibility of worms with *pmk-1* RNAi (Figure 4A). The results indicate that the p38 MAPK pathway is epistatic to *riok-1* while the other immune pathways are not (Figure S4 in Supplementary Material). Further tests using mutant worms of the p38 MAPK-signaling pathway showed similar results. The knockdown of *riok-1* in p38 MAPK *pmk-1* (Figure 4B) and MAPKK *sek-1* (Figure 4C) mutants led to hypersensitivity to *A. dhakensis*. The *nsy-1(ums8)* (a p38 MAPK gain-of-function allele)-mutant worms treated with *riok-1* RNAi did not gain additional resistance to *A. dhakensis* infection (Figure 4D). *A. dhakensis* AAK1 induced the activation of the p38 MAPK pathway based on the evidence of p38 phosphorylation in a Western blot analysis (Figure 4E). In addition, the knockdown of *riok-1* induced p38 phosphorylation (Figure 4E) and the increased expression of an array of p38 MAPK downstream genes (Figures 4F–J). These results suggest that *riok-1* lies upstream of the p38 MAPK pathway and that it also plays a role in negative regulation of this pathway.

### RIOK-1 Feeds Back Negatively to the p38 MAPK Pathway Through Transcriptional Factor *skn-1* After *A. dhakensis* Infection in *C. elegans*

**Figure 5:**
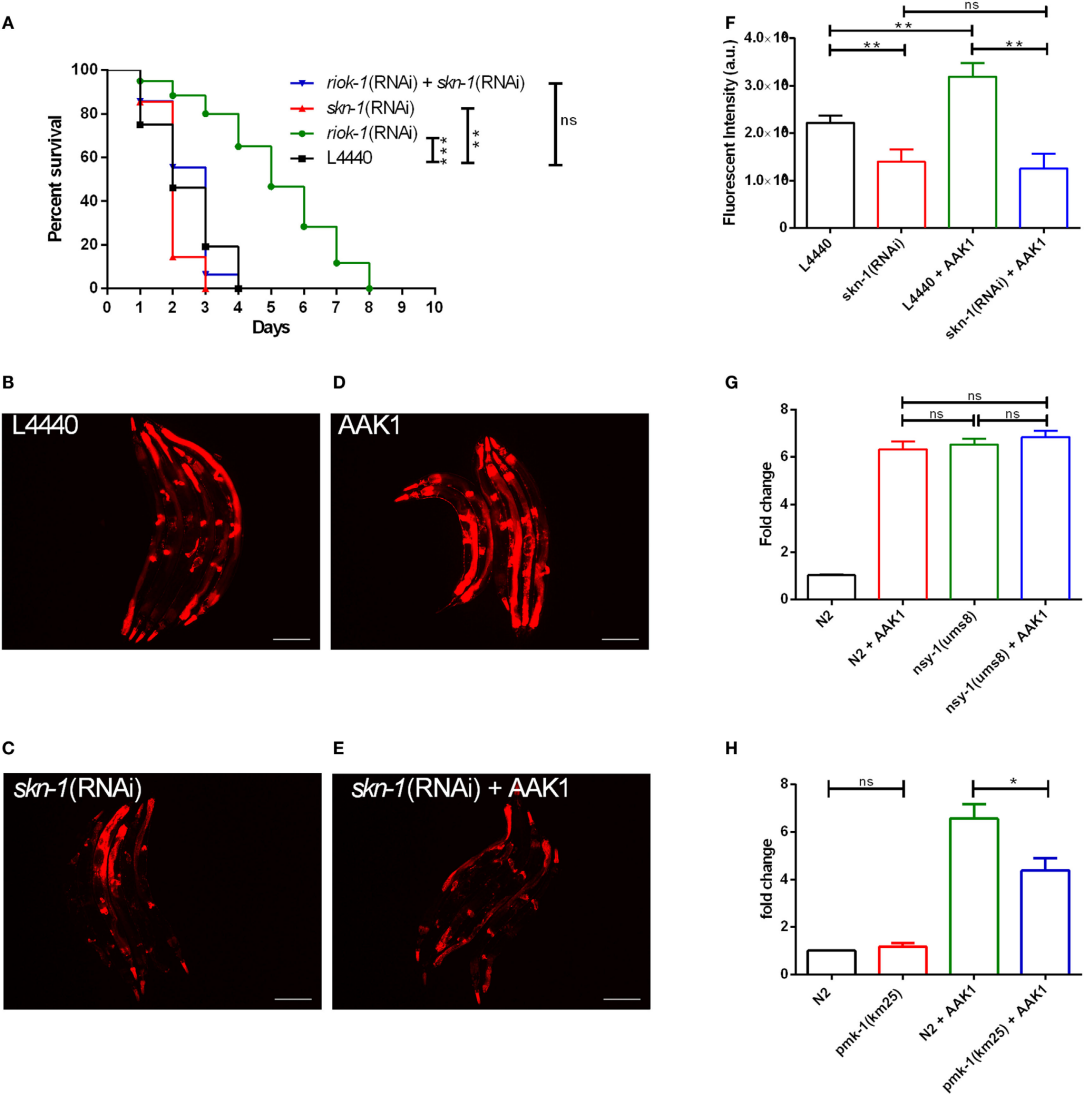
The increase in *riok-1* is *skn-1*-dependent in *Caenorhabditis elegans* with *Aeromonas dhakensis* infection. **(A)** Single knockdown of *skn-1* or simultaneous knockdown of *skn-1* and *riok-1* resulted in a decrease in the life span in *C. elegans* with *A. dhakensis* infection. **(B)** *riok-1* expression in *riok-1* transcriptional reporter worms with RNAi control vector L4440. **(C)** *riok-1* expression in *riok-1* transcriptional reporter worms with RNAi knockdown of *skn-1*. **(D)** *riok-1* expression in *riok-1* transcriptional reporter worms with *A. dhakensis* AAK1 infection. **(E)** *riok-1* expression in *riok-1* transcriptional reporter worms with RNAi knockdown of *skn-1 and A. dhakensis* AAK1 infection. **(F)** A summary of the florescence quantification of **(B–E)**. **(G)** The expression level of *riok-1* as determined using quantitative real time-PCR (qRT-PCR) in the p38 gain-of-function-mutant worms *nsy-1(ums8*) with and without *A. dhakensis* AAK1 infection was similar to those in wild-type N2 worms with *A. dhakensis* AAK1 infection. **(H)** The expression levels of *riok-1* as determined using qRT-PCR in the p38 MAPK/*pmk-1*-mutant worms *pmk-1(km25)* with *A. dhakensis* AAK1 infection were lower than in wild-type N2 worms with *A. dhakensis* AAK1 infection. The scale bars in **(B–E)** are all 100 µm. ***P < 0.001, **P < 0.01, and *P < 0.05.

Previous studies have shown that p38 MAPK downstream gene *skn-1*, a transcription factor that is able to bind to the promoter region of *riok-1*, promotes *riok-1* expression in the intestines of *C. elegans* (32). SKN-1 has also been proven to be an important component in the intestinal immune system of worms (37). Our results were comparable with a previous finding indicating that *riok-1* expression is affected by the p38 MAPK downstream transcription factor *skn-1* (32). Worms with a suppressed *riok-1* expression had more resistance after *A. dhakensis* infection in contrast to those treated with *skn-1* RNAi. By contrast, the enhancement of resistance to *A. dhakensis* infection mediated by *riok-1* knockdown was decreased once *skn-1* was suppressed (Figure 5A). Taken together, these results suggest that *skn-1*, the p38 MAPK downstream gene, is also epistatic to *riok-1*. Consistent with the findings reported by Mendes et al. (32), the intestinal florescence in *riok-1* transcriptional reporter worms was decreased when the worms were treated with *skn-1* RNAi (Figure 5C), in contrast to the RNAi control with the RNAi empty vector L4440 (Figure 5B). The expression of *riok-1* in the transcriptional reporter worms co-treated with *skn-1* RNAi and *A. dhakensis* was similar to those treated with *skn-1* RNAi alone (Figure 5E) and was lower than that found in the control (Figure 5D). The quantification of florescence intensity in *riok-1* transcriptional reporter worms under different conditions is summarized (Figure 5F). In addition, the expression level of *riok-1* in the p38 MAPK *nsy-1(ums8)* gain-of-function mutants was similar to those in the wild-type N2 worms and was not increased additionally by *A. dhakensis* in the *nsy-1(ums8)* worms (Figure 5G). The results also indicated that *A. dhakensis* could not induce *riok-1* expression directly without passing through the p38 MAPK-signaling pathway. In addition, the *riok-1* expression level induced by *A. dhakensis* AAK1 infection in the wild-type N2 worms was significantly higher than those in the *pmk-1(km25)* mutants, suggesting the coexistence of another pathway that is independent of p38 MAPK-regulated activation of *riok-1* expression (Figure 5H). Briefly, the results show that *A. dhakensis*-induced *riok-1* expression occurs partially through the p38 MAPK pathway. The activation of the pathway is *skn-1*-dependent, and *riok-1* suppresses immunity by targeting the p38 MAPK pathway, participating in a feedback circuit to regulate the immune system in *C. elegans*.

### RIOK-1 Deficiency in *C. elegans* Increases the Susceptibility to Different Gram-Negative Bacterial Infections

**Figure 6:**
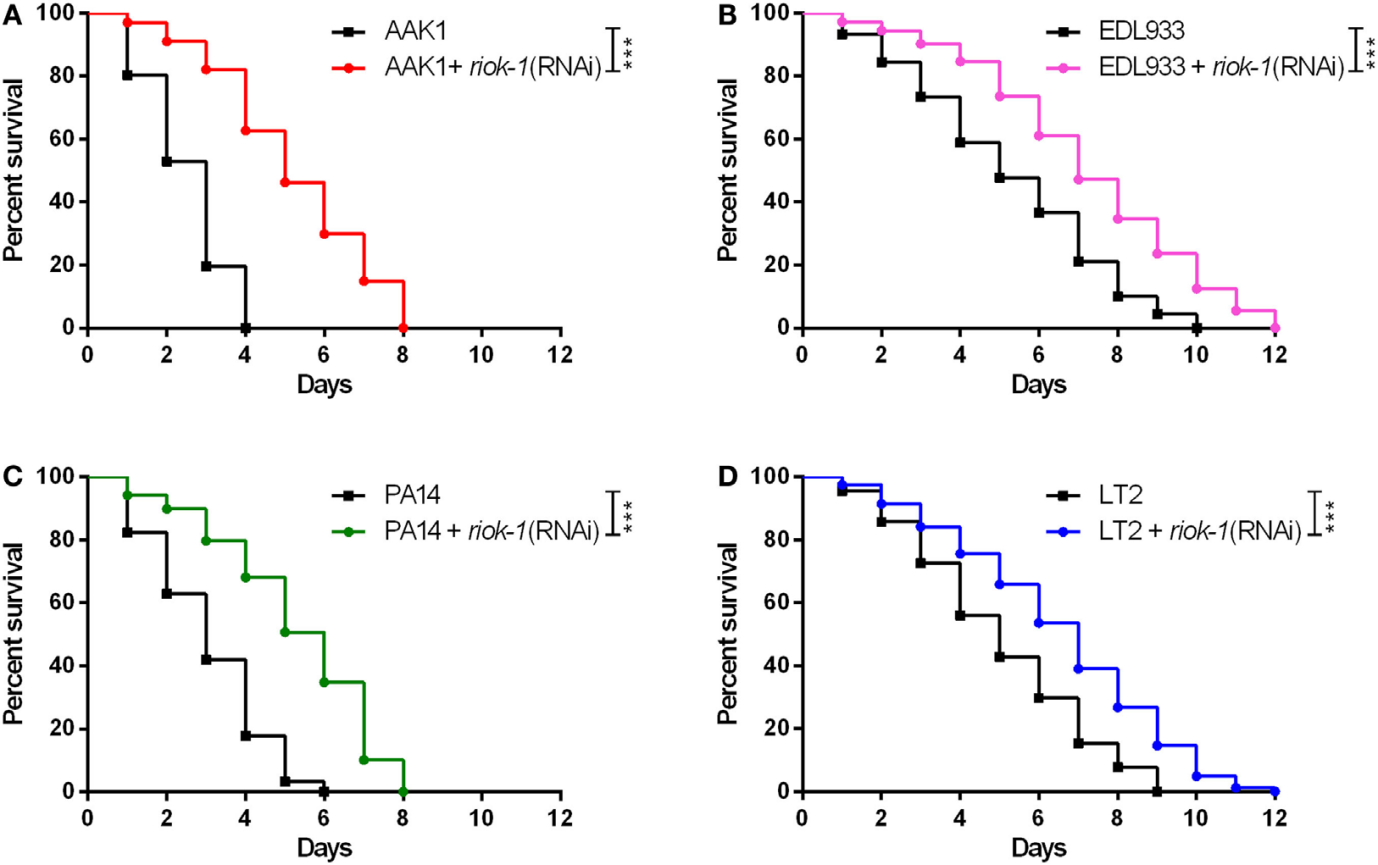
Suppressed expression of *riok-1* increases the resistance to pathogens in *Caenorhabditis elegans*. Knockdown of *riok-1* with RNAi in *C. elegans* confers resistance to **(A)** *Aeromonas dhakensis* AAK1, **(B)** enterohemorrhagic *Escherichia coli* EDL933, **(C)** *Pseudomonas aeruginosa* PA14, and **(D)** *Salmonella typhimurium* LT2. The statistical analyses of pathogens with and without *riok-1* RNAi are all ***P < 0.001.

Our study proved *riok-1* to be a negative regulator of the p38 MAPK immune pathway in *C. elegans*. In order to understand whether the *riok-1-*p38 MAPK pathway is a universal mechanism against bacterial infection, resistance to different bacterial species, including *Salmonella typhimurium* (38, 39), enterohemorrhagic *E. coli* (26), and *P. aeruginosa* (23, 40, 41), which have been reported to activate the p38 MAPK pathway in *C. elegans*, was studied. Our survival analysis showed that *riok-1* knockdown mediated by RNAi in *C. elegans* conferred resistance not only to *A. dhakensi*s AAK1 (Figure 6A) but also to enterohemorrhagic *E. coli* EDL933 (Figure 6B), *P. aeruginosa* PA14 (Figure 6C), and *S. typhimurium* LT2 (Figure 6D). The results demonstrated that the *riok-1*–p38 MAPK pathway plays an important role in immunity against Gram-negative bacterial infection in *C. elegans*.

**Figure 7:**
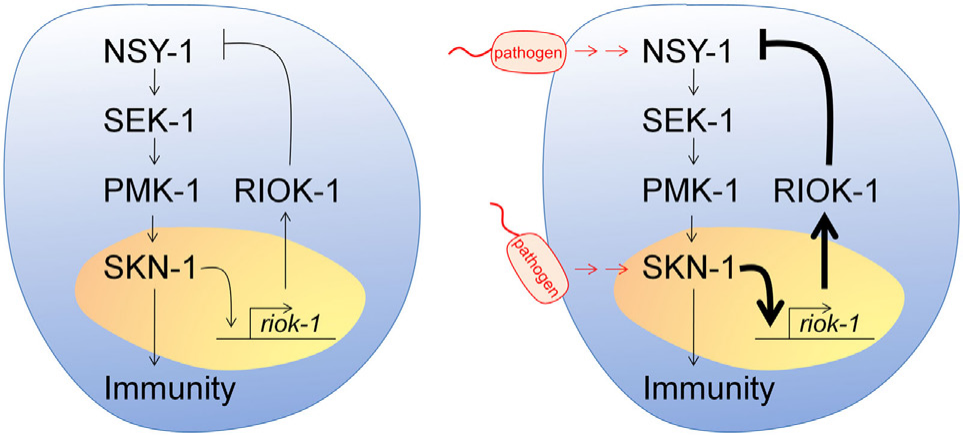
Model of a p38 MAPK, SKN-1, and RIOK-1 feedback loop upon infection in *Caenorhabditis elegans*. Pathogens activate the p38 MAPK pathway and increase the expression of *riok-1* through the transcription of *skn-1*. RIOK-1 has an important role in a feedback loop that regulates the expression of the p38 MAPK pathway in order to maintain immune homeostasis.

According to our results, we propose a negative feedback loop model among p38 MAPK, SKN-1, and RIOK-1 in *C. elegans* (Figure 7). *A. dhakensis* infection activates the p38 MAPK pathway in *C. elegans*. It also induces *riok-1* expression by the p38 MAPK pathway through the transcription of *skn-1*, and *riok-1* suppresses the p38 MAPK pathway in a feedback loop in order to maintain immune homeostasis.

## Discussion

In this study, we screened the kinases that may participate in a defensive mechanism against *Aeromonas* infection in a focused RNAi kinome library in *C. elegans* (8, 9, 11). RIOK-1 as a negative regulator of immunity against infection conferred resistance to *Aeromonas* in *C. elegans* when it was suppressed. The resistant group may include those acting as pathogen-dependent receptors or suppressors of host immunity, such as *riok-1*. By contrast, candidate genes might be associated with enhancers of immunity against infection and will make worms hypersensitive to infection when their expression is suppressed (Table 1B).

From an evolutionary perspective, immune homeostasis is an important survival strategy, particularly for creatures such as bacterivorous nematodes that live in environments rich with microbes that must differentiate pathogens from potential food sources. Improper activation of immune responses in humans underlies many disorders, such as inflammatory bowel disease, cancer, and autoimmune diseases (3, 42). A mechanism of feedback control for a hyperactivated immune pathway has been discovered in flies and *C. elegans* (4, 7, 43). Here, we discovered that *riok-1* is a novel immune suppresser of the infection-activated p38 MAPK pathway in *C. elegans* and plays an important role in achieving immune homeostasis.

A previous study showed that the aberrant activation of p38 MAPK-dependent immune responses is toxic to *C. elegans* (23). Feedback control of hyperactivation of the p38 immune pathway to avoid negative physiological consequences has been reported. For example, MEMO-1 was complexed with RHO-1/RhoA/GTPase and loss of *memo-1* resulted in enhanced interaction of RHO-1 with BLI-3/NADPH oxidase, thereby stimulating ROS production that signals the p38 MAPK pathway to promote stress resistance and longevity (4). In another example, infection was associated with an increase in p38 MAPK/PMK-1 activity (5), which was negatively regulated by MAPK phosphatase VHP-1 (7). However, the knockdown of *vhp-1* with RNAi did not increase resistance to *A. dhakensis* when compared with the control in our study (Figure S5 in Supplementary Material). The results suggested that the p38 MAPK-negative regulator *vhp-1*, although important in *P. aeruginosa* PA14 infection (7), may not participate in resistance against *A. dhakensis* infection in *C. elegans*. Herein, we identified *riok-1*, which regulates the p38 MAPK pathway in a pattern distinguished from *memo-1* and *vhp-1*. However, the exact mechanism through which *riok-1* functions as a suppressor of the p38 MAPK immune pathway remains unclear and requires further study.

The expression sites of *riok-1* suggest where *riok-1* regulates immunity against infection. Our research demonstrated that *riok-1* is expressed in neurons and in the intestines. Previous studies have shown that neurons can modulate the innate immune response of intestine cells in response to bacterial infection in *C. elegans* (44). The neuronal products modulated the *daf-2*/*daf-16* insulin-like pathway in the intestines of *C. elegans*. By contrast, no epistatic relationship between *riok-1* and *daf-2* was discovered in our study (Figure S4A in Supplementary Material). In addition, the knockdown of *skn-1* decreased the expression of *riok-1* predominately in the intestine (Figure 5C), indicating that the p38 MAPK–*skn-1* immune pathway is not operative in neurons.

In summary, we discovered that *riok-1* is a novel innate immune suppressor and proposed a negative feedback model among the p38 MAPK, SKN-1, and RIOK-1 loop in *C. elegans* in order to maintain immune homeostasis. Further study of RIOK-1 may bring new insights into defects in the p38 MAPK pathway underlying human diseases and may reveal new therapeutic opportunities for bacterial infection.

## Author Contributions

P-LC, Y-WC, W-CK, and C-SC conceived and designed the experiments. Y-WC performed all the experiments. Y-WC, P-LC, and C-SC analyzed the data. Y-WC, C-SC, and P-LC wrote the paper. C-SC, W-CK, and P-LC reviewed and edited the paper.

## Conflict of Interest Statement

The authors declare that the research was conducted in the absence of any commercial or financial relationships that could be construed as a potential conflict of interest.
