# Supplementary material for: RIOK-1 Is a Suppressor of the p38 MAPK Innate Immune Pathway in Caenorhabditis elegans
Source: Front Immunol. 2018 Apr 17;9:774. doi: 10.3389/fimmu.2018.00774 (PMC5913292; doi:10.3389/fimmu.2018.00774)
Supplement: Supplementary file 2 [file Data_Sheet_1.DOCX]

***Supplementary Material***

**RIOK-1 is a suppressor of the p38 MAPK innate immune pathway in *Caenorhabiditis elegans***

**Yi-Wei Chen, Wen-Chien Ko, Chang-Shi Chen^*^, and Po-Lin Chen^*^**

**^*^Correspondence:**

Chang-Shi Chen: cschen@mail.ncku.edu.tw

Po-Lin Chen: cplin@mail.ncku.edu.tw

**Supplementary Figures and Tables**

**Supplementary Table 1.** Screening of the RNAi kinome library of *C. elegans* identified a host gene against pathogen *Aeromonas dhakensis* AAK1. All the data were repeated in triplicate.





**Supplementary Figure 2.** Supplementary data for Figure 3. (A) *riok-1* is expressed in the pharynx (p), intestines (i), spermatheca (s), and pan-neuronally (n) in *riok-1* transcriptional reporter worms. (B) *riok-1* is expressed in the pharynx (p), intestines (i), spermatheca (s), and pan-neuronally (n) in translational reporter worms. (C) The expression site of *riok-1* in pharynx is mainly in the pharyngeal neuron, which is covered by pharyngeal muscle (marker: *myo-2p::GFP*). (D) The expression site of *riok-1* in the vulva is mainly in the vulva neuron, which is not co-localized with vulva muscle (marker: *myo-3p::GFP*). (E) The tissue specificity of tissue-specific RNAi worm strains with an intestine-specific *act-5* RNAi clone, a neuron-specific *unc-73* RNAi clone, and an epidermis-specific *bli-1* RNAi clone were summarized with occurrence of phenotypes in corresponding worm strains. The scale bars in (A) and (B) are all 100 μm.





**Supplementary Figure** **3.** Progeny deficiency is not associated with *A. dhakensis* resistance mediated by *riok-1*deletion in *C. elegans*. The lifespans of *glp-4(bn2)* worms showed no difference from those observed in N2 strains with or without *riok-1* knockdown mediated by RNAi after *A.* *dhakensis* infection.





**Supplementary Figure 4.** Screen of the well-known immune pathways epistatic to *riok-1.* Lifespan of *C. elegans* with simultaneous knockdown of the target gene and *riok-1* using RNAi. (A) *daf-2* of the insulin-like signaling pathway (B) *lgg-1* of autophagy (C) *dbl-1*of the TGF-β signaling pathway (D) *mpk-1* of the *mpk-1* MAPK pathway (E) *mek-2* of the MAPK/ERK pathway (F) *nipi-3* of the *nlp-29*-MAPK pathway.


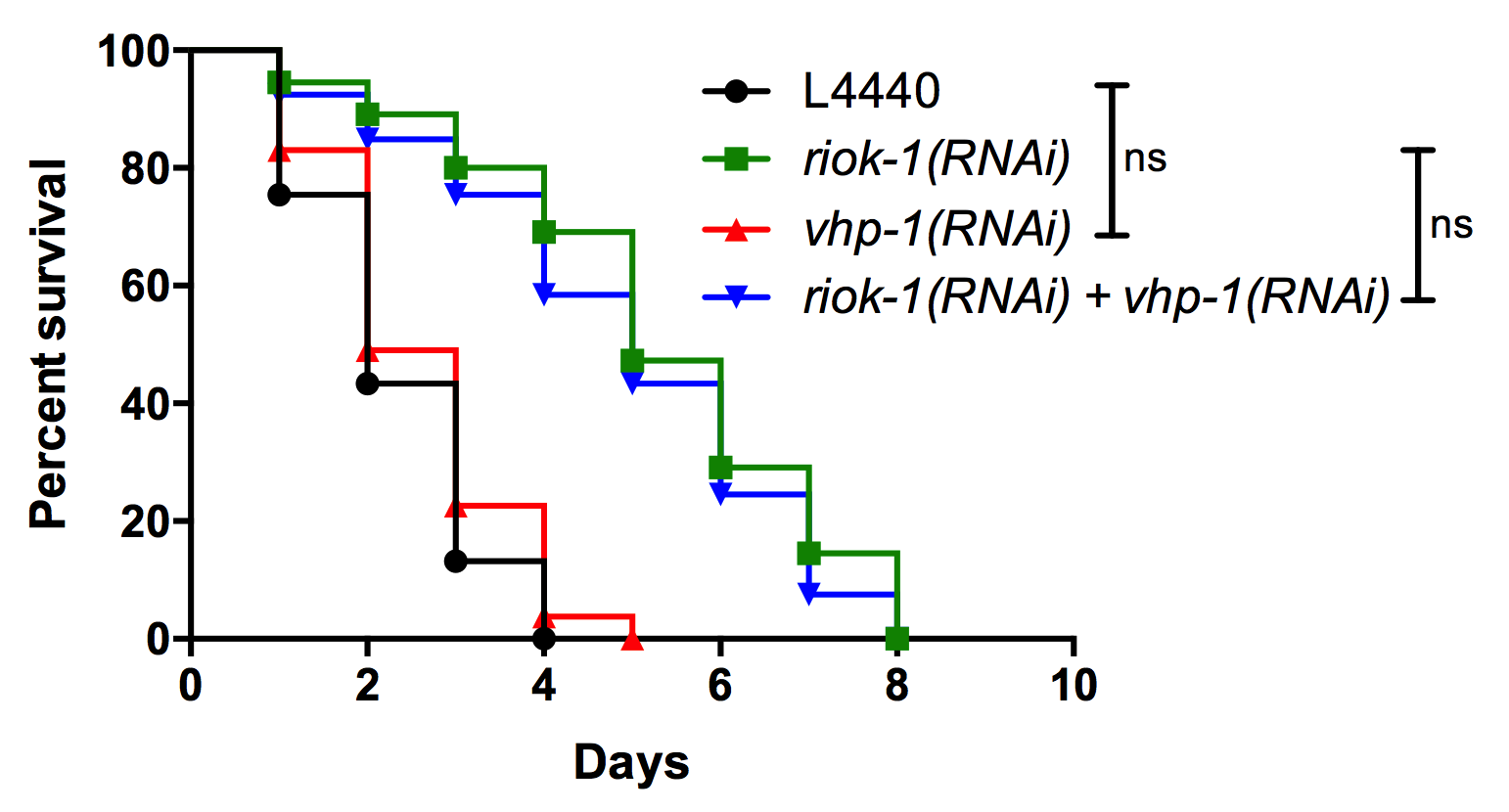


**Supplementary Figure** **5.** *C. elegans* tyrosine-protein phosphatase *vhp-1* is not involved in the p38 MAPK- RIOK-1 signaling pathway upon *A. dhakensis* infection. Double RNAi knockdown of *riok-1* and *vhp-1* showed resistance to *A. dhakensi*s; however knockdown of *vhp-1* with RNAi singly did not increase the resistance of the infected worms.


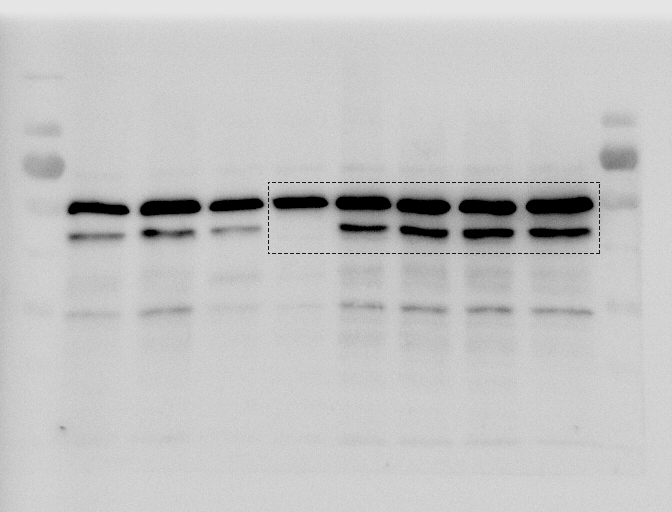


**Supplementary Figure** **6.** Unprocessed data of western blot analysis shown as Figure 4E.
